# INTEREST GROUP SESSION—QUALITATIVE RESEARCH: INNOVATIVE QUALITATIVE AND MIXED-METHODS STUDIES OF OLDER ADULTS’ LIVED EXPERIENCES IN NETWORKS OF CARE AND CARING

**DOI:** 10.1093/geroni/igz038.2041

**Published:** 2019-11-08

**Authors:** Allyson M Washburn, Abby Schwartz

**Affiliations:** 1 National University, La Jolla, California, United States; 2 East Carolina University, School of Social Work, Greenville, North Carolina, United States

## Abstract

Researchers are challenged both when querying older adults about their lived experience and later when analyzing these rich interview data to characterize and preserve their truths. This symposium presents innovative strategies to meet these challenges in ways that deepen our understanding of older adults’ lives within their networks of care and caring. Participants in two interpretive phenomenological studies were family caregivers. One examined the impact of the Virtual Dementia Tour® on participants’ perceptions of their family member’s lived experience of dementia, identifying changed realities and approaches to caregiving following their experience. The second analyzed in-depth interviews of aging caregivers about future care for their adult children diagnosed with autism spectrum disorders, finding that they had difficulty identifying caregiving support for the future while feeling the need to make plans and decisions now. Two mixed methods studies explored older adults’ experience of their current environment. In the first, measures of social cognition and engagement informed the interpretation of combined phenomenological-hermeneutic and conventional content analyses of nursing home residents’ responses to questions about their relationships, finding that they valued day-to-day social interactions as connections with longtime friends were maintained. The second study combined focus groups, in-depth interviews, and web-based surveys during the psychometric testing of the Person-Place Fit Measure for Older Adults to assess older adults’ experience of aging in a particular place with people they find important. The strategies detailed in these four papers transformed more conventional approaches to deepen our understanding of older adults’ experiences of their lives with others.

